# Calcium alterations signal either to senescence or to autophagy induction in stem cells upon oxidative stress

**DOI:** 10.18632/aging.101130

**Published:** 2016-12-08

**Authors:** Aleksandra V. Borodkina, Alla N. Shatrova, Pavel I. Deryabin, Anastasiia A. Griukova, Polina A. Abushik, Sergei M. Antonov, Nikolay N. Nikolsky, Elena B. Burova

**Affiliations:** ^1^ Department of Intracellular Signaling and Transport, Institute of Cytology, Russian Academy of Sciences, St. Petersburg, 194064, Russia; ^2^ Laboratory of Comparative Neurophysiology, Sechenov Institute of Evolutionary Physiology and Biochemistry, Russian Academy of Sciences, St. Petersburg, 194223, Russia

**Keywords:** endometrial stem cells, senescence, autophagy, calcium, oxidative stress

## Abstract

Intracellular calcium ([Ca^2+^]_i_) has been reported to play an important role in autophagy, apoptosis and necrosis, however, a little is known about its impact in senescence. Here we investigated [Ca^2+^]_i_ contribution to oxidative stress-induced senescence of human endometrium-derived stem cells (hMESCs). In hMESCs sublethal H_2_O_2_-treatment resulted in a rapid calcium release from intracellular stores mediated by the activation of PLC/IP3/IP3R pathway. Notably, further senescence development was accompanied by persistently elevated [Ca^2+^]_i_ levels. In H_2_O_2_-treated hMESCs, [Ca^2+^]_i_ chelation by BAPTA-AM (BAPTA) was sufficient to prevent the expansion of the senescence phenotype, to decrease endogenous reactive oxygen species levels, to avoid G0/G1 cell cycle arrest, and finally to retain proliferation. Particularly, loading with BAPTA attenuated phosphorylation of the main DNA damage response members, including ATM, 53BP1 and H2A.X and reduced activation of the p53/p21/Rb pathway in H_2_O_2_-stimulated cells. Next, we revealed that BAPTA induced an early onset of AMPK-dependent autophagy in H_2_O_2_-treated cells as confirmed by both the phosphorylation status of AMPK/mTORC1 pathway and the dynamics of the LC3 lipidization. Summarizing the obtained data we can assume that calcium chelation is able to trigger short-term autophagy and to prevent the premature senescence of hMESCs under oxidative stress.

## INTRODUCTION

Calcium considered being an incredibly multifaceted ion that is implicated in various biological functions, including protein secretion, exocytosis, contraction, gene transcription and cell growth [1]. Remarkably, any disturbance in intracellular calcium homeostasis can provoke a switch from normal regulation of cell function to a signal for cell death [2, 3]. For now, the central role of calcium deregulation is well established in the induction of apoptosis and necrosis [1, 4, 5]. Alternatively to death, cells encountering certain stress may cope with it by inducing either senescence or autophagy [6, 7]. Several studies [8-12] previously reported calcium implication in senescence progression. However, complete picture still remains unclear.

Cellular senescence is a physiologic response directed to prevent the propagation of damaged cells [13]. Typically it is elicited by replicative exhaustion or by a variety of stresses causing DNA damage [14]. Senescence is characterized by a permanent cell cycle arrest and a subsequent loss of proliferative capacity. Though senescent cells remain metabolically and transcriptionally active, they undergo dramatic alterations in morphology, extensive changes in gene expression, and acquire a distinctive secretory phenotype, which affects the tissue homeostasis [13, 15]. Furthermore, senescent cells display unrepaired DNA damages that persistently activate the ATM/ATR-dependent DNA damage response (DDR) pathway, which, in turn, leads to the activation of p53, the up-regulation of p21 and cell cycle arrest at G1/S transition. It is now widely accepted that senescence is involved in tumor suppression, aging, multiple pathologies, wound healing and normal embryonic development [16, 17].

Going back to the possible role for calcium in senescence progression, it should be noted that several reports indicated elevation of intracellular calcium levels during oncogene-, rotenone-induced as well as replicative senescence [8, 9, 12]. However, the main focus in these studies was made on the role of mitochondrial calcium accumulation as an underlying cause of enhanced reactive oxygen species (ROS) production and altered mitochondrial function in senescent cells. The other authors mentioned the interplay between calcium and transcription factor p53 in the context of senescence, suggesting that cellular alterations underlying p53 activation might be associated with calcium homeostasis [11, 18]. Nevertheless, to date there is no clear evidence about the exact relationship between p53 and calcium.

Another pivotal cellular stress response along with senescence is a lysosomal delivery pathway, termed autophagy. This process is commonly defined as an evolutionary conserved catabolic pathway by which damaged cellular proteins and organelles are delivered to lysosomes for degradation and recycling [19]. Autophagy can enable adaptation to stress by removing protein aggregates and damaged organelles, thus maintaining cellular homeostasis and promoting cellular viability [7, 11, 20]. Although there are undeniable argues in favor of intracellular calcium involvement in autophagy regulation [21-23], yet there is no consensus regarding the direct role of calcium in this process. It is assumed that Ca^2+^ signaling may have opposite effects in normal versus stressed cells and thus differently control basal (suppressing) versus augmented (promoting) autophagic activity in response to stress [22].

Human endometrium-derived mesenchymal stem cells (hMESCs) are an easily available source of adult stem cells [24]. Mounting evidence suggest that these cells can be successfully utilized in regenerative medicine [25, 26]. According to our previous data, hMESCs via activation of the canonical ATM/Chk2/p53/p21/Rb pathway enter the premature senescence in response to sublethal oxidative stress [27], what may limit the effectiveness of their potential clinical application. This observation highlights the importance of understanding the complex nature of senescence and signaling pathways of its induction. In human stem cells calcium signaling is mentioned primarily with regard to their differentiation potential [28, 29]. A particular emphasis is made on the investigation of calcium level modulation during transformation of non-excitable undifferentiated stem cells to excitable cell types, such as neurons and muscles. For now, it is postulated that Ca^2+^-mediated signaling is essential for the stemness maintenance as well as for promoting development and differentiation of stem cells [28]. Nevertheless, the role of intracellular calcium in stem cell stress responses remains poorly elucidated.

To sum up, on the one hand, it is clear that oxidative stress-induced senescence of human endometrial stem cells may dramatically diminish the effectiveness of their transplantation. On the other hand, all above highlights the existing gap between calcium signaling and senescence progression. Fulfilling this gap may provide a strategy to enhance the effectiveness of hMESCs clinical application. In this regard, the present study aimed to reveal the impact of intracellular calcium on oxidative stress-induced senescence of hMESCs.

## RESULTS

### Oxidative stress induces a rapid [Ca^2+]^i increase in hMESCs

**Figure 1:**
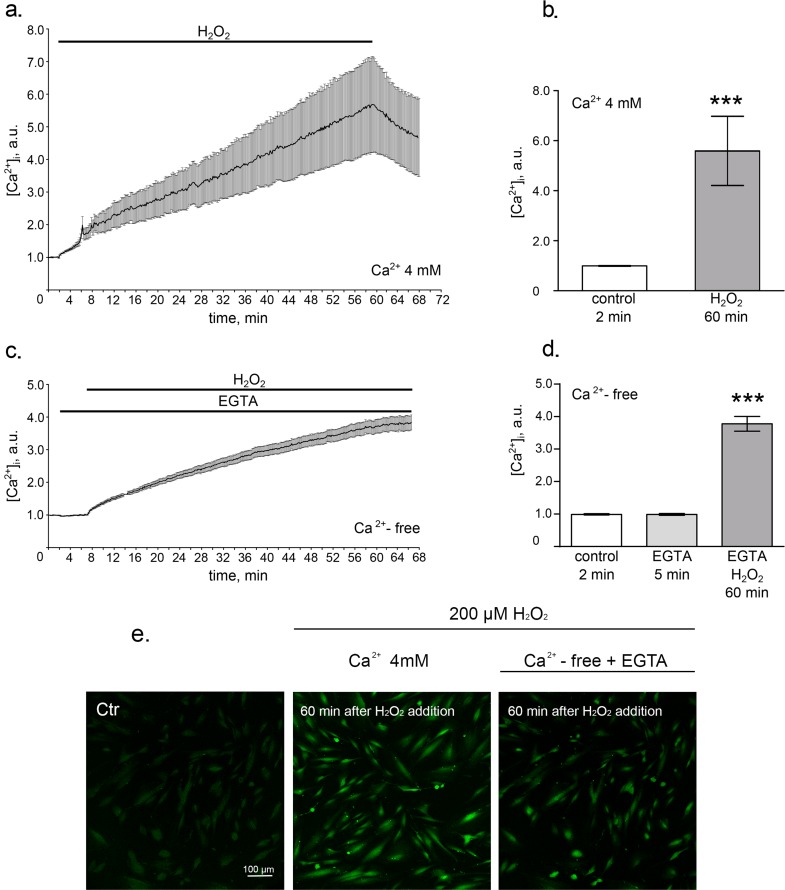
Oxidative stress induces intracellular calcium increase hMESCs were treated with 200 μM H_2_O_2_ for 1 h and intracellular calcium levels were determined using Fluo-3 imaging techniques. (**a**) Time course of the relevant increase of [Ca^2+^]_i_ during 1 h H_2_O_2_ treatment in Ca^2+^-containing solution. Number of cells = 24. (**b**) Histogram, based on the data from (a), reflecting the relevant values of [Ca^2+^]_i_ on 2 min in control and on 60 min after H_2_O_2_ addition. (**c**) Time course of the relevant increase of [Ca^2+^]_i_ during 1 h H_2_O_2_ treatment in Ca^2+^-free solution containing 4mM EGTA. Number of cells = 28. (**d**) Histogram, based on the data from (**c**), reflecting relevant values of [Ca^2+^]_i_ in Ca^2+^-free basic solution on 2 min in control, on 5 min of EGTA action and on 60 min of H_2_O_2_ action. (**e**) Confocal images of hMESCs loaded with Fluo-3AM on 2 min of control, on 60 min of H_2_O_2_ action in Ca^2+^-containing solution and on 60 min of H_2_O_2_ action in Ca^2+^-free solution with EGTA. Scale bar is 100 μm and valid for all images. Values are M ± Std.Er. *** p<0.0001 by Mann-Whitney test. Application intervals and duration are marked with black lines above the graphs. Ctr – untreated cells. Representative results of three independent experiments are shown.

We first investigated the effects of the sublethal oxidative stress on the intracellular calcium levels in hMESCs. Using Fluo-3 imaging techniques we revealed a rapid elevation of [Ca^2+^]_i_ in response to H_2_O_2_ addition that gradually increased during 1 h treatment (Fig. 1a, 1b and 1e). It is well known that cytosolic calcium influx may originate from both intra- and extracellular sources [30]. Thus, to discriminate the wellspring of the observed [Ca^2+^]_i_ elevation in H_2_O_2_-treated hMESCs, we performed assays in the absence of extracellular calcium. The results presented in Fig. 1c, 1d and 1e show that omitting Ca^2+^ from bathing solution had no dramatic suppressive effect on [Ca^2+^]_i_ increase in response to H_2_O_2_ stimulation. Therefore, Ca^2+^ release from intracellular stores rather than Ca^2+^ entry across the plasma membrane might be responsible for the [Ca^2+^]_i_ elevation in hMESCs during H_2_O_2_ treatment.

### PLC/IP3/IP3R pathway orchestrates the prompt cytosolic Ca^2+^ response in H_2_O_2_-treated hMESCs

**Figure 2:**
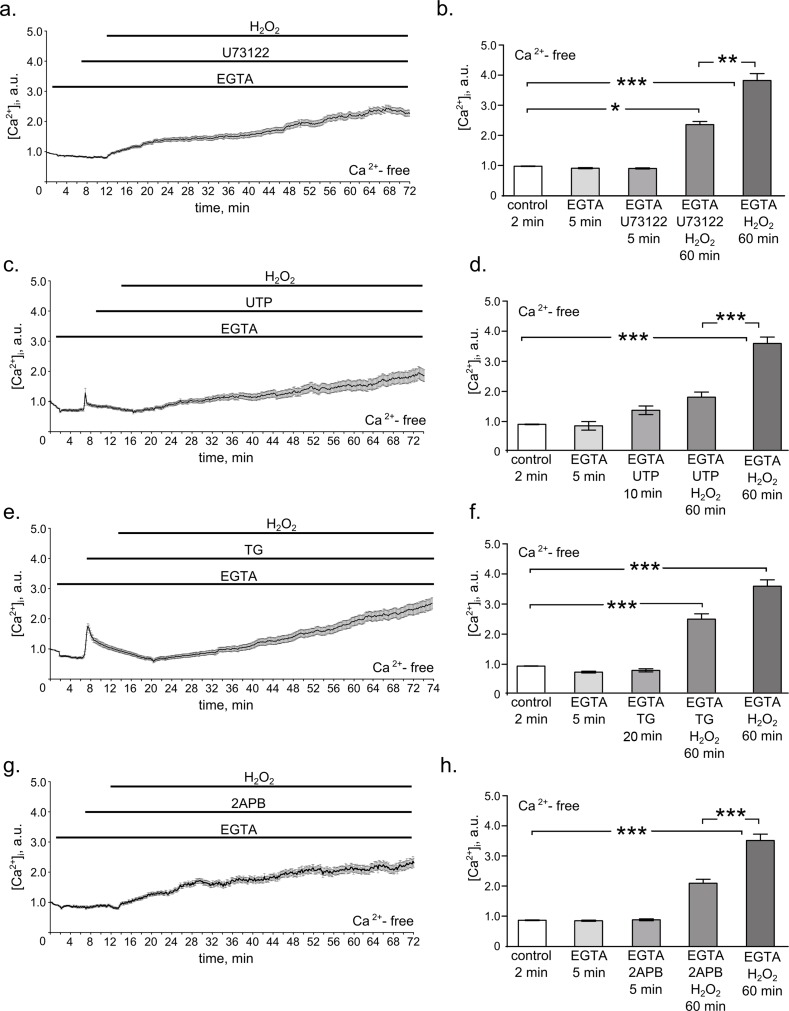
PLC/IP3/IP3R pathway mediates H_2_O_2_-induced intracellular calcium elevation in hMESCs All measurements were done in Ca^2+^-free basic solution supplemented with 4 mM EGTA in 2 min after the beginning of the imaging, which further was present throughout the whole experiment. (**a**) Time course of the relevant [Ca^2+^]_i_ increase during 5 min of 1 μM U73122 pretreatment followed by 60 min of 200 μM H_2_O_2_ stimulation. (**b**) Histogram based on the data from **(a**), reflecting the relevant values of [Ca^2+^]_i_ on 2 min in control, on 5 min of EGTA alone action, on 5 min of U73122 action, on 60 min of U73122 + H_2_O_2_ action, and on 60 min of H_2_O_2_ alone action. Number of cells = 24. (**c**) Time course of the relevant [Ca^2+^]_i_ increase in H_2_O_2_-stimulated hMESCs pretreated with 100 μM UTP for 10 min. (**d**) Histogram, based on the data from (c), reflecting the relevant values of [Ca^2+^]_i_ on 2 min in control, on 5 min of EGTA alone action, on 10 min of UTP action, on 60 min UTP + H_2_O_2_ action, and on 60 min of H_2_O_2_ alone action. Number of cells = 29. (**e**) Time course of the relevant [Ca^2+^]_i_ increase during 20 min of 1 μM TG pretreatment followed by 60 min of 200 μM H_2_O_2_ stimulation. (**f**) Histogram based on the data from (**e**), reflecting the relevant values of [Ca^2+^]_i_ on 2 min in control, on 5 min of EGTA alone action, on 20 min of TG action, on 60 min TG + H_2_O_2_ action, and on 60 min of H_2_O_2_ alone action. Number of cells = 31. (**g**) Time course of the relevant [Ca^2+^]_i_ increase in H_2_O_2_-stimulated hMESCs pretreated with 50 μM 2-APB for 5 min. (**h**) Histogram based on the data from (**g**), reflecting the with relevant values of [Ca^2+^]_i_ on 2 min in control, on 5 min of EGTA alone action, on 5 min of 2-APB action, on 60 min 2-APB + H_2_O_2_ action, and on 60 min of H_2_O_2_ alone action. Number of cells = 28. Results are shown as M ± Std.Er. *p<0.05, **p<0.001, ***p<0.0001 by Mann-Whitney test. Application intervals and duration are marked with black lines above the graphs. Representative results of three independent experiments are shown.

Having established the fact of the [Ca^2+^]_i_ increase in hMESCs during 1 h H_2_O_2_ treatment, we next explored what signaling pathway might be responsible for it. As a matter of fact Ca^2+^ release from internal stores occurs primarily from the endoplasmic reticulum (ER) [30-32]. Thus, we speculate that inositol trisphosphate (IP3), known to mediate a rapid calcium store release through the activation of IP3 receptors (IP3R) in the ER membrane, might be a good candidate for generating calcium increase in H_2_O_2_-treated hMESCs. In order to check this hypothesis we applied various drugs to modulate IP3 synthesis and determined cytosolic Ca^2+^ signals with Fluo-3 in H_2_O_2_-treated cells. The phospho-lipase C (PLC) is the up-stream regulator of the intracellular IP3 generation [33]. Therefore, to investigate whether PLC-dependent IP3 production could contribute to the revealed [Ca^2+^]_i_ increase in response to H_2_O_2_, we examined the effects of its inhibitor U-73122 in the Ca^2+^-free external solution containing EGTA. Interestingly, blocking IP3 synthesis with U-73122 distinctly suppressed H_2_O_2_-induced Ca^2+^ increase (Fig. 2a and 2b).

To further verify the involvement of PLC/IP3 pathway in H_2_O_2_-induced [Ca^2+^]_i_ elevation, we utilized the uridine triphosphate (UTP) as the modulator of PLC activation. In various cells UTP was shown to elicit initial release of Ca^2+^ from IP3-sensitive stores, leading to ER pool depletion [22, 34]. As expected, hMESCs pretreatment with UTP in Ca^2+^-free external solution supplied with EGTA resulted in a rapid release of Ca^2+^ into cytosole. In this case subsequent H_2_O_2_ addition had a lesser effect on [Ca^2+^]_i_ levels compared to H_2_O_2_-stimulated cells non-pretreated with UTP, indicating the essential role of PLC-mediated store depletion in response to H_2_O_2_ (Fig. 2c and 2d).

As an additional confirmation of the ER stored calcium contribution to the hMESCs oxidative stress response, we next applied a non-competitive inhibitor of the Ca^2+^ ATPase (SERCA) localized in the ER – thapsigargin (TG) [22, 35]. By inhibiting SERCA, TG blocks the ability of the cell to pump calcium into the ER store, what leads to gradual calcium outflow from the cell. Thus, by utilizing very different mechanisms of action both compounds TG and UTP ultimately lead to the ER pool depletion. However, in contrast to an immediate calcium outflow caused by UTP, TG induced a comparatively slow calcium release. Results presented in Fig. 2e and 2f generally are in accordance with the data obtained in presence of UTP: cell pretreatment with TG in Ca^2+^-free external solution supplied with EGTA slightly attenuated H_2_O_2_-induced calcium increase. This is the extra evidence in support of the ER-dependent calcium release in hMESCs upon the oxidative stress.

It is well documented that IP3 causes an immediate calcium release through the activation of IP3R in the ER membrane. Thus, to confirm that IP3R might be responsible for the mobilization of intracellular Ca^2+^ stores, we next blocked the activation of IP3R using antagonist of these receptors 2-aminoethoxydiphenyl borate (2-APB) to reduce Ca^2+^ release from the intracellular stores [36]. Interestingly, 2-APB significantly diminished H_2_O_2_-induced [Ca^2+^]_i_ rise (Fig. 2g and 2h). The obtained data clearly indicate that observed intracellular calcium elevation in H_2_O_2_-stimulated cells at least in part is mediated by the activation of PLC/IP3/IP3R pathway.

### Intracellular calcium accumulation mediates H_2_O_2_-induced senescence of hMESCs

**Figure 3:**
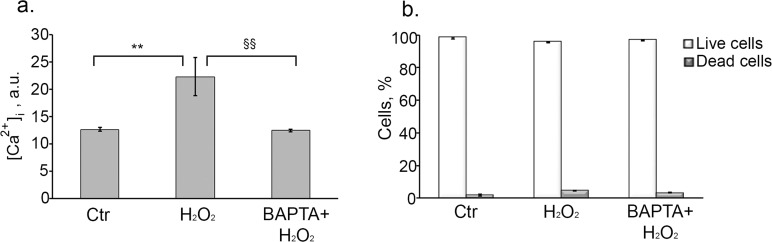
Loading cells with BAPTA effectively decreases H_2_O_2_-induced [Ca^2+^]_i_ elevation, and has no effect on cell viability Cells were either pretreated or not with 10 μM BAPTA (loading procedure is described in “Materials and Methods” section), then were subjected to 200 μM H_2_O_2_ for 1 h with the following H_2_O_2_ replacement and cell cultivation under normal conditions for the indicated time. (**a**) Intracellular calcium levels measured by FACS after staining hMESCs with the fluorescent probe Fluo-3AM at day 6 after the oxidative stress. (**b**) Application of 10 μM BAPTA had no effect on viability of H_2_O_2_-treated hMESCs. The percentage of viable cells was evaluated in 24 h after H_2_O_2_ treatment by FACS analysis as described in “Materials and Methods” section. M ± Std.dev., N=3. **p<0.005, versus control; §§p<0.005, versus H_2_O_2_-treated cells by Student's t-test. Ctr – untreated cells.

We next wanted to know whether the observed increase in [Ca^2+^]_i_ has an impact on H_2_O_2_-induced senescence development. To this end, firstly we measured intracellular calcium level at day 6 after senescence induction. As shown in Fig. 3a senescent cells displayed about 2 times higher basal Ca^2+^ as compared to control cells, suggesting that Ca^2+^ might be involved in senescence progression. In order to confirm this assumption we applied widely used intracellular calcium chelator – BAPTA-AM. Loading cells with 10 μM BAPTA-AM had no effect on cell viability (Fig. 3b), but effectively reduced [Ca^2+^]_i_ in H_2_O_2_-treated cells up to the control levels at day 6 (Fig. 3a).

**Figure 4:**
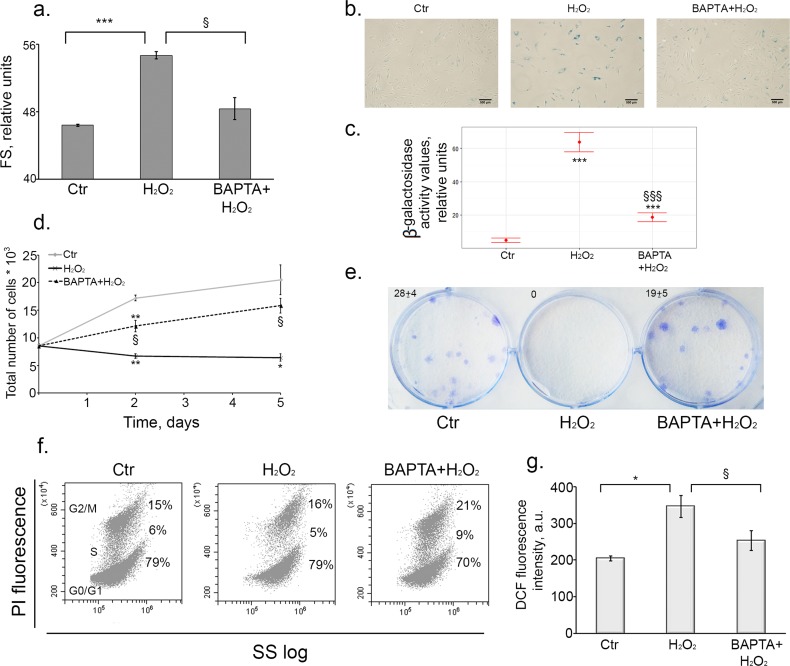
Intracellular calcium chelation by BAPTA prevents oxidative stress-induced senescence of hMESCs Cells were treated as indicated in the legend to Figure 3. (**a**) BAPTA partially prevented H_2_O_2_-induced increase of cell size. Cell size was determined at day 6 after the oxidative stress. Forward scatter (FS) reflects the average cell size. (**b**) SA-β-Gal staining of untreated, H_2_O_2_-treated and (BAPTA+H_2_O_2_)-treated hMESCs. In 5 days after the oxidative stress cells were harvested by trypsinization and plated at a density of 4.5*10^3^ cells per cm^2^ and additionally cultured for 5 days, in order to perform staining in non-confluent cultures. Scale bar is 500 μm and valid for all images. (**c**) Quantification of β-galactosidase activity values in control, H_2_O_2_-treated and (BAPTA+H_2_O_2_)-treated hMESCs. (**d**) BAPTA retained cell proliferation as compared to H_2_O_2_-treated cells. Cell number was determined by FACS at indicated time points. (**e**) BAPTA pretreated hMESCs maintained the colony forming ability. In 14 days after oxidative stress cells were fixed and stained to monitor cell growth. (**f**) [Ca^2+^]_i_ chelation in H_2_O_2_-treated resulted in cell cycle phase re-distribution. Flow cytometric analysis of cell cycle phase distribution: the percentage of cells in the G0/G1, S, and G2/M phases, visualization of phase distribution is based on light-scattering analysis. SS log - side scattering. (**g**) Intracellular ROS levels detected at day 6 after H_2_O_2_ stimulation by FACS analysis after staining with H_2_DCFDA. Images shown are representative of experiments performed at least three times. Graphs are presented as M ± Std.dev., and the Student's t-test was used to determine p-value. *p<0.05, **p<0.005, ***p<0.001, versus control; §p<0.05, §§§p<0.001, versus H_2_O_2_-treated cells. Ctr – untreated cells.

Earlier we have convincingly shown that hMESCs treated with 200 μM H_2_O_2_ enter the premature senescence accompanied by the irreversible cell cycle arrest, cell hypertrophy and enhanced SA-β-Gal staining [37]. First of all we checked whether calcium chelation affected oxidative stress-induced senescence. As presented in Fig. 4a, 4b and 4c BAPTA application impeded H_2_O_2_-induced increase of the cell size as well as SA-β-Gal activity, indicating some modulation of the senescence phenotype. Moreover, BAPTA treatment led to marked increase in the number of proliferating cells compared to H_2_O_2_-stimulated cells, indicating that Ca^2+^ chelation overcame the growth arrest induced by H_2_O_2_ (Fig. 4d and 4e). Additional evidence in favor of growth arrest escape in BAPTA-loaded cells was obtained by the analysis of the cell cycle phase distribution. In our previous findings we clearly showed that hMESCs treatment with 200 μM H_2_O_2_ led to the prolonged irreversible cell cycle arrest in all phases. [37]. Loading cells with BAPTA resulted in a distinct phase redistribution and enhanced amount of cells in S-phase, as can be seen in two-parametric histogram based on the distribution analysis with using light scattering (Fig. 4f).

Moreover, the above results were verified by two extra sets of the experiments. First set was performed on H_2_O_2_-treated hMESCs preloaded with another well-known [Ca^2+^]_i_ chelator – Quin2-AM. The second one was directed to prevent H_2_O_2_-induced senescence of human embryonic fibroblasts pretreated with BAPTA. In both cases [Ca^2+^]_i_ chelation resulted in partial prevention of oxidative stress-induced senescence, strongly confirming calcium implication in senescence progression (Suppl. Fig. 1a, 1b, 1c and Suppl. Fig. 2a, 2b, 2c, 2d).

As we described earlier, senescent hMESCs are characterized by persistently elevated ROS levels [27]. To test whether calcium chelation could alter ROS production, we evaluated intracellular ROS levels in BAPTA-loaded and unloaded senescent cells, using H_2_DCFDA staining. Loading of H_2_O_2_-stimulated cells with BAPTA led to a noticeable decrease in the intracellular ROS levels (Fig. 4g).

Summarizing all described above results, we can conclude that H_2_O_2_-induced increase of intracellular calcium levels is implied in hMESCs senescence progression, whereas calcium chelation may protect the cells from premature senescence.

### Loading with BAPTA attenuates DNA damage response activation in H_2_O_2_-treated cells

**Figure 5:**
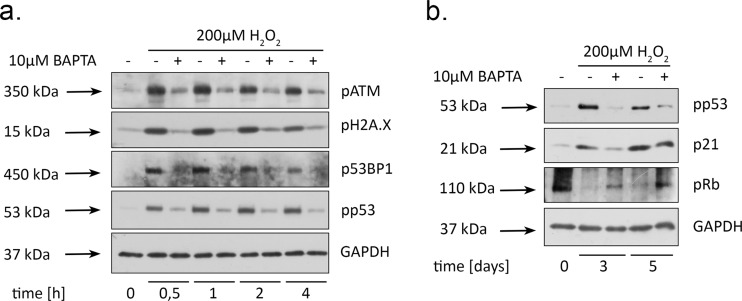
BAPTA attenuates activation of both DNA damage response and p53/p21/Rb pathway in H_2_O_2_-treated hMESCs Cells were treated as indicated in the legend to Figure 3 and were subsequently analyzed by western blotting with the indicated antibodies at the various time points. (**a**) Phosphorylation levels of the main DDR members: ATM, H2A.X, 53BP1, as well as p53. **(b**) Western blot analysis of p53 and Rb phosphorylation, and p21 protein expression performed at indicated time points. Representative results of the three experiments are shown in the Figure. GAPDH was used as loading control.

Our previous data clearly state that premature senescence in H_2_O_2_-treated hMESCs develops by the following scenario: added H_2_O_2_ rapidly penetrates into the cells and causes DNA damage as detected by the activation of the main DNA damage response (DDR) members, including ATM, 53BP1 and H2A.X. DDR activation, in turn, leads to the initiation of the p53/p21/Rb pathway, cell cycle arrest and further senescence progression [27]. Taking into account the prompt intracellular calcium response to H_2_O_2_ stimulation, further we speculated that buffering of [Ca^2+^]_i_ by BAPTA might affect an early DDR. Indeed, loading with BAPTA dramatically reduced phosphorylation of each DDR participant as compared to H_2_O_2_-treated cells over the entire observation period (Fig. 5a). Accordingly, in H_2_O_2_-treated hMESCs, calcium chelation significantly attenuated phospho-rylation of p53, prevented enhanced p21 protein expression and elevated the Rb phosphorylation levels long after senescence induction (Fig. 5b). These findings correlate well with the partial proliferation retaining of (BAPTA+H_2_O_2_)-treated hMESCs (Fig. 4d and 4e). These results were confirmed by applying Quin2-AM in hMESCs (Suppl. Fig. 1d, e). Interesting-ly, in BAPTA-pretreated fibroblasts we also detected reduction of ATM and H2A.X prosphoryation as well as decreased activity of p53/p21 pathway, but the observed effects were less pronounced, what might be due to cell specificity (Suppl. Fig. 2e, f). Taken together, these data suggest that BAPTA-induced DDR down-regulation is implicated in the senescence prevention in stressed hMESCs.

### BAPTA triggers autophagy in hMESCs under the oxidative stress

**Figure 6:**
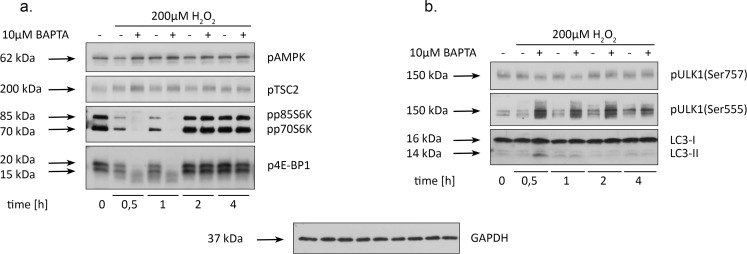
BAPTA induces an AMPK-dependent autophagy in H_2_O_2_-treated hMESCs Cells were treated as indicated in the legend to Figure 3 and were subsequently analyzed by western blotting with the indicated antibodies. (**a**) Western blot analysis of pAMPK, pTSC2, p70S6K and p4E-BP1 at the various time points after H_2_O_2_ addition. (**b**) Alterations in ULK1 phosphorylation at Ser757 and Ser555 as well as LC3 lipidization induced by BAPTA pretreatment in H_2_O_2_-stimulated cells. Representative results of the three experiments are shown in the Figure. GAPDH was used as loading control.

Recent reports revealed that AMPK-dependent autophagy may protect cells from oxidative stress-induced senescence [38]. We next explored whether the observed protective effect of calcium chelation from H_2_O_2_-induced senescence might be interlinked with autophagy process. The most common mechanism underlying the AMP-activated protein kinase (AMPK)-mediated autophagy progression is suppression of the mTORC1 pathway: activated AMPK phosphorylates TSC2, what increases the GAP activity of TSC2 and, thus reduces mTORC1 activation [39]. As it turned out, BAPTA pretreatment caused a short-term increase in AMPK activation in 30 min after H_2_O_2_ stimulation (Fig. 6a). Accordingly, loading cells with BAPTA promoted a rapid increase in pTSC2 and a reduction in phosphorylation levels of the main mTORC1 targets – p70S6K and 4E-BP1 as compared to H_2_O_2_-treated cells (Fig. 6a). Another important protein involved in the initiation of autophagosome formation is Unc-51-like kinase 1 (ULK1) [40]. Notably, ULK1 is a down-stream target for both AMPK and mTOR, however, they oppositely regulate its activity: AMPK phosphorylates ULK1 at Ser555, thus activating it, whereas mTOR is responsible for ULK1 downregulation by phosphorylat-ing it at Ser757 [40]. Fig. 6b displays enhanced phosphorylation of “pro-autophagic” Ser555 of ULK1 and attenuated “anti-autophagic” phosphorylation of ULK1 at Ser757 in H_2_O_2_-treated hMESCs in presence of BAPTA. Finally, we were able to detect the appearance of a lipidated form of LC3 (LC3-II) and its further turnover (Fig. 6b), what serves as a crucial evidence in favor of autophagy process in BAPTA-loaded hMESCs under the oxidative stress. It should be noted that activation kinetics of mTOR signaling and ULK1 protein in (Quin2-AM)-pretreated hMESCs was similar to those of BAPTA-pretreated but was delayed in time (Suppl. Fig. 1f). Minor differences in effects of BAPTA and Quin2-AM are possibly connected with the fact that BAPTA more rapidly binds intracellular calcium ions as compared to other known chelators. However, the data obtained on fibroblasts were rather contradictory: loading H_2_O_2_-stimulated fibroblasts with BAPTA simultaneously led to the opposite results in mTOR signaling (enhanced phosphorylation of 4E-BP1 and altered phospho-rylation of p70S6K) and in signaling for autophagy (increased phosphorylation of both pro- and anti-autophagic sites of ULK1) (Suppl. Fig. 2g). These differences between hMESCs and fibroblasts might be connected with cell specificity. Overall, we can assume that [Ca^2+^]_i_ chelation by BAPTA partially prevents oxidative stress-induced senescence of hMESCs via autophagy initiation.

## DISCUSSION

In keeping with the latter observations oxidative stress may induce DNA damage, autophagy, premature senescence and Ca^2+^ mobilization, however, yet there is no common opinion whether and how these responses are interrelated [27, 37, 41-43]. In the present study we investigated the role of intracellular calcium in oxidative stress-induced senescence progression in hMESCs.

According to the literary data, calcium outflow from intracellular pools occurs principally from ER wherein one of the most important Ca^2+^ release channels – IP3R – has been identified [30-32]. The ligand for IP3R is IP3 that binds to the receptor, thus leading to calcium release from ER to cytoplasm [52]. Actually, different exogenously added oxidants can stimulate IP3R-mediated Ca^2+^ mobilization [53-56]. Indeed, we revealed that in H_2_O_2_-treated hMESCs intracellular calcium release was provided through IP3R. Typically, IP3R is regulated by the signaling pathways mediating IP3 generation [30-32, 57], the most common is initiated by PLC that catalyzes the hydrolysis of phosphatidylinositol-4,5-bisphosphate to diacylglycerol and IP3 at the plasma membrane [33]. In context of hMESCs, sublethal H_2_O_2_ treatment led to an increased intracellular calcium levels namely via PLC/IP3/IP3R pathway. In contrast, in intraneurons of the spinal cord H_2_O_2_ provoked calcium outflow through IP3R, but application of the phospholipase C (PLC) inhibitor was not able to reduce elevation of cytoplasmic calcium [58]. This observation allowed authors to suggest that the targets of H_2_O_2_ action lie downstream in the IP3 signaling cascade.

To date, only a few studies elucidating calcium implication in cellular senescence have been published [8, 9, 12, 38]. The common notion is that intracellular calcium increases during senescence progression. For example, McCarthy and coauthors reported a higher basal calcium levels in replicatively senescent fibroblasts as compared to non senescent cells [8]. Another group evidenced that cytosolic Ca^2+^ rise is important for rotenone-induced senescence in human neuroblastoma cells [9]. Our observations are in accordance with the described above, as we also revealed an elevation of cytoplasmic calcium in H_2_O_2_-stimulated hMESCs, suggesting that Ca^2+^ might be involved in senescence progression. In favor of this hypothesis calcium chelation by BAPTA partially prevented senescence in hMESCs, as detected by both the proliferation maintaining and partial loss of the senescence phenotype.

Earlier we have shown that permanently enhanced ROS are typical for the senescent hMESCs [27]. Remarkably, calcium chelation was able to decline endogenous ROS levels long after hMESCs senescence induction. For now, it is well established that calcium released from the ER through IP3R is then taken up by mitochondria [30, 59]. Thereby, sustained intracellular calcium increase during senescence may lead to mitochondrial calcium accumulation and altered mitochondrial activity, what mediates elevated ROS generation [12]. This may have sense in context of senescent hMESCs, as previously we have displayed that an increased mitochondrial activity is responsible for long-term ROS production [27]. In line with this assumption, french research group recently postulated implication of the mitochondrial calcium accumulation in both replicative and oncogene-induced senescence [12]. Moreover, decreasing calcium levels by mitochondrial calcium uniporter knockdown allowed cells to escape senescence.

It is generally accepted that the most prominent feature of senescent cells is irreversible cell cycle arrest, resulting in proliferation block [13-15]. Notably, BAPTA pretreatment decreased the activity of p53/p21/Rb pathway and thus prevented proliferation loss in H_2_O_2_-treated hMESCs. There are some data suggesting that intracellular Ca^2+^ fluctuations may modulate the functioning of this signaling cascade [60]. For instance, Ca^2+^ can regulate the ability of Ca^2+^-binding proteins to interact with p53, leading to its enhanced transcriptional activity or protein stability [11]. It was also demonstrated, that Ca^2+^/calmodulin may influence cell cycle progression via cdc2 kinase activation and Rb phosphorylation [61].

Our previous findings indicate that functional activation of the p53/p21/Rb pathway in hMESCs occurs as a result of DNA damage caused by H_2_O_2_ treatment [27]. Here we revealed that calcium chelation significantly decreased phosphorylation of the ATM, H2A.X and 53BP1 proteins in H_2_O_2_-treated hMESCs. Similar data were obtained by other researchers: intracellular calcium chelation by Quin2-AM suppressed DNA damage induced by either H_2_O_2_ or ROS produced by xanthine/xanthine oxidase [62, 63]. The initial studies suggested that the protective effects of calcium chelators against oxidative stress-initiated DNA damage might be associated with their ability to chelate iron ions as well [63, 64]. Iron ions are known to catalyze Fenton reaction with production of highly reactive OH. radicals which enhance DNA damage [65]. Worth mentioning that in the cited articles authors applied rather high concentrations of calcium chelators as well as comparatively long pretreatment time. However, the more recent observations pointed out that even a very high concentration of BAPTA failed to chelate iron in intramitochondrial pool [66]. Moreover, it was reported that BAPTA retained the protective effect even being added after the stress [67]. Although our results do not prove or disapprove the capacity of BAPTA to chelate iron ions, it definitely should be taken into consideration. According to the modern point of view, a so-called “connecting link” between stress-induced Ca^2+^ mobilization and DNA damage might be PARP1 activation [67]. On the one hand, PARP1 functions as a DNA damage sensor that binds to both single and double strand breaks, and serves as a platform for the recruitment of proteins associated with the DDR [68]. Thereby, PARP1 participates in a variety of DNA damage consequences, including chromatin remodeling, transcriptional regulation, DNA repair, cell cycle arrest, senescence, or cell death. On the other hand, it was shown that PARP1 activation was mediated by an increase in intracellular Ca^2+^ caused either by β-lapachone-, H_2_O_2_-, or peroxynitrite-treatment [67, 69].

Therefore, we can speculate that calcium chelation by BAPTA may prevent PARP1 activation and decrease DDR initiation, thereby averting cell cycle arrest and senescence progression in H_2_O_2_-treated hMESCs. In support of this hypothesis it was shown that intracellular calcium chelation was able to preclude both PARP1 activation and γH2A.X foci formation and a subsequent oxidative stress-induced cell death [67].

A growing number of studies are focused on the investigation of the interplay between senescence and autophagy [38, 70-72]. For example, genetic impairment of autophagy in young satellite cells caused entry into senescence, whereas re-establishment of the process reversed senescence and recovered regenerative functions of these cells [72]. The same is true for the oxidative stress conditions: autophagy restoration averted H_2_O_2_-induced senescence in fibroblasts [38]. Based on the described above, we proposed that BAPTA-induced senescence prevention in H_2_O_2_-treated hMESCs might somehow be connected to the autophagic process.

It is well known that Ca^2+^ homeostasis is tightly linked to the autophagy, but depending on the cellular state, Ca^2+^ alterations may either inhibit or stimulate autophagy [22, 23, 73]. Interestingly, various Ca^2+^-mobilizing agents (thapsigargin, ATP and ionomycin) were clearly shown to induce autophagy, indicating pro-autophagic role of increased cytosolic calcium levels [74, 75]. However, it should be noted that Ca^2+^ ionophores as well as TG-induced depletion of ER, described in the above articles, elevate Ca^2+^ to a non-physiological means and for prolonged periods of time, whereas physiological [Ca^2+^]_i_ signals typically have shorter duration and smaller amplitudes [23]. Another evidence considering calcium as an activator of autophagy is based on the observations that calcium chelation decreased stress-induced autophagy in various cells [22, 76, 77].

Despite of the intracellular calcium mobilization detected in our stress conditions sublethal H_2_O_2_ treatment did not cause any detectable autophagy in hMESCs. Contrarily, calcium chelation by BAPTA resulted in rapid short-term autophagy stimulation, as indicated by the appearance of the lipidated LC3 form. Similar inhibitory role of Ca^2+^ towards autophagy was described for TKO cells, where Ca^2+^ signals were abolished by either IP3R knocking down or its suppression [32]. Furthermore, we revealed that BAPTA-triggered autophagy was AMPK-dependent. AMPK – a major inducer of autophagy – is activated in response to decrease in ATP and a following increase in AMP or ADP [78, 79]. As it was mentioned above, calcium outflowing from ER is then uptaken by mitochondria, where it can drive ATP production via regulation of mitochondrial dehydrogenases and ATP synthase [59, 23]. Decreased calcium level may impair mitochondrial bioenergetics thus elevating AMP/ATP ratio and leading to AMPK activation [23]. Accordingly, it was shown that different manipulations, including inhibition of IP3R-mediated Ca^2+^ release or IP3R activity (by xestospongin B), inhibition PLC (by U73122), attenuation of mitochondrial Ca^2+^ uptake (Ru360 or mitochondrial calcium uniporter RNAi) etc., resulted in reduced ATP production followed by AMPK activation and the induction of pro-survival autophagy [23, 32]. These data allow assuming that in H_2_O_2_-treated hMESCs BAPTA-induced senescence prevention might be linked with the disturbance in Ca^2+^ transfer from ER to mitochondria, leading to activation of AMPK, which in turn activates autophagy as a survival mechanism.

**Figure 7:**
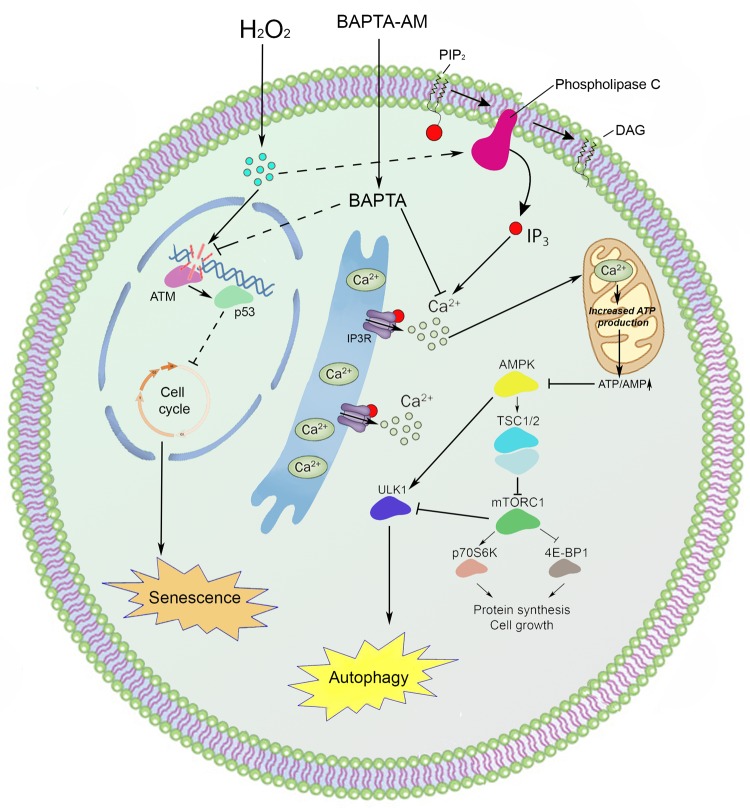
An interaction scheme displays the proposed molecular mechanism of intracellular calcium involvement in the premature senescence and autophagy of hMESCs under oxidative stress Various oxidants, including t-butylhydroperoxide, menadione, H_2_O_2_, thimerosal, and superoxide generating systems such as hypoxanthine/xanthine oxidase, are shown to cause an elevation of cytosolic Ca^2+^ in intact cells [1, 44-48]. Our findings strongly support these observations: in hMESCs we revealed an increase in intracellular calcium levels in response to sublethal H_2_O_2_ treatment. Depending on the concen-tration and type of oxidant used, intracellular calcium rise can be mediated either by Ca^2+^ import from the extracellular spaces and/or calcium release from internal stores, such as ER [1, 41, 49]. For example, high H_2_O_2_ concentrations lead to a sustained elevation of cytosolic calcium, which was not observed in Ca^2+^-free medium, suggesting that severe oxidative stress evoked Ca^2+^ uptake from extracellular spaces [50]. On the contrary, mild oxidative stress caused by moderate H_2_O_2_ levels increased cytoplasmic Ca^2+^ in smooth muscle and endothelial cells even in calcium-free medium, but it failed to do so if Ca^2+^ was first depleted from the ER [50, 51]. Results presented in our study are in line with the last notion: (1) H_2_O_2_-induced mobilization of intracellular calcium persisted even in Ca^2+^-free bathing solution, (2) application of either UTP or TG, known to deplete ER store, greatly attenuated observed Ca^2+^ increase; together indicating that in hMESCs sublethal oxidative stress triggered calcium release predominantly from internal stores.

In conclusion, the present study is the first to elucidate the impact of intracellular calcium mobilization in oxidative stress-induced senescence of hMESCs. Here we revealed that calcium chelation can protect cells from premature senescence by both the reduction of DDR activation with a subsequent decrease in p53/p21/Rb pathway functioning and the autophagy stimulation (Fig. 7).

## MATERIALS AND METHODS

### Cell culture

Human mesenchymal stem cells were isolated from desquamated endometrium in menstrual blood from healthy donors (hMESCs, line 2304) as described previously [80]. hMESCs have a positive expression of CD73, CD90, CD105, CD13, CD29, and CD44 markers and absence of expression of the hematopoietic cell surface antigens CD19, CD34, CD45, CD117, CD130, and HLADR (class II). Multipotency of isolated hMESCs is confirmed by their ability to differentiate into other mesodermal cell types, such as osteocytes and adipocytes. Besides, the isolated hMESCs partially (over 50 %) express the pluripotency marker SSEA-4 but do not express Oct-4. Immunofluorescent analysis of the derived cells revealed the expression of the neural precursor markers nestin and beta-III-tubulin. This suggests a neural predisposition of the established hMESCs. These cells are characterized by high rate of cell proliferation (doubling time 22–23 h) and high cloning efficiency (about 60 %). Human embryonic lung fibroblasts were obtained from the Research Institute of Influenza (St. Petersburg, Russia). Both cell lines cultured in complete medium DMEM/F12 (Gibco BRL, MD, USA) supplemented with 10 % FBS (HyClone, USA), 1 % penicillin-streptomycin (Gibco BRL, MD, USA) and 1 % glutamax (Gibco BRL, MD, USA) at 37°C in humidified incubator, containing 5 % CO_2_. Cells were harvested by trypsinization and plated at a density of 15*10^3^ cells per cm^2^ for hMESCs and 37*10^3^ cells per cm^2^ for fibroblasts. To avoid complications of replicative senescence, cells at early passages (5-9 for hMESCs and 15-20 for fibroblasts) were used in all experiments.

### Loading of Fluo-3 AM and Ca^2+^ imaging

Cells were loaded with 10 μM Fluo-3 AM (Life Technologies, CA, USA) using conventional protocols as recommended by the manufacturer. In brief, hMESCs were incubated with Fluo-3 AM for 60 min in Ca^2+^-free basic solution (144 mM NaCl, 4 mM KCl, 0.33 mM NaH_2_PO_4_, 5.5 mM glucose, 0.53 mM MgCl_2_, 10 mM HEPES (Sigma-Aldrich, MO, USA), pH adjusted to 7.4 using NaOH) in the dark at room temperature. Then, Fluo-3 AM was washed out, and cells were incubated in the external basic solution with 4 mM CaCl_2_ (Sigma-Aldrich, MO, USA) for another 15 min in the dark. Coverslips with Fluo-3-loaded cultures were placed in the perfusion chamber, which was mounted on the stage of Leica TCS SP5 MP inverted microscope (Leica Microsystems, Germany). Fluorescence was activated with 488 nm laser light and emission was measured within the wavelength range from 500 to 560 nm. Images were captured every 2.5 seconds during ∼ 60 min experiments.

In Ca^2+^ imaging experiments we used basic solution containing 144 mM NaCl, 4 mM KCl, 0.33 mM NaH_2_PO_4_, 5.5 mM glucose, 4 mM CaCl_2_, 0.53 mM MgCl_2_, 10 mM HEPES, pH adjusted to 7.4 using NaOH. In Ca^2+^-free experiments we used the same basic solution without CaCl_2_. In order to avoid Ca^2+^ contamination in Ca^2+^-free experiments, basic solution was additionally supplemented with 4 mM EGTA (Sigma-Aldrich, MO, USA).

### Oxidative stress conditions

H_2_O_2_ stock solution in PBS was prepared from 30 % H_2_O_2_ (Sigma- Aldrich, MO, USA) just before adding. Cells were treated with 200 μM H_2_O_2_ for 1 h, after that washed twice with PBS to remove H_2_O_2_, and re-cultured in fresh complete medium for various durations as specified in individual experiments.

### Experimental design

In this study, the several types of cell treatments were applied depending on the assay. All assays were performed in Ca^2+^-free bath solution supplied with 4 mM EGTA, to exclude the impact of extracellular calcium. To suppress PLC activity, 1 μM U-73122 (Calbiochem, CA, USA) was added 5 min prior H_2_O_2_-treatment. In order to induce ER calcium release, cells were pretreated with either 100 μM UTP (Sigma-Aldrich, MO, USA) for 10 min or 1 μM TG (Sigma-Aldrich, MO, USA) for 20 min. In order to block the activation of IP3R, we utilized treatment with 50 μM 2-APB (Sigma-Aldrich, MO, USA) for 5 min before oxidative stimulation. To chelate intracellular Ca^2+^ cells were first loaded with 10 μM BAPTA-AM (Sigma-Aldrich, MO, USA) or 1 μM Quin2-AM (SERVA, NY, USA) for 30 min in Ca^2+^-free solution and then additionally incubated for 20 min in Ca^2+^-free solution in the absence of the AM ester to allow intracellular de-esterification, followed by H_2_O_2_ treatment.

### FACS analysis of cell viability, cell size cell cycle progression

Adherent cells were rinsed twice with PBS and harvested by trypsinization. Detached cells were pelleted by centrifugation. Finally, detached and adherent cells were pooled and resuspended in PBS. 50 μg/ml propidium iodide (PI) was added to each sample just before analysis and mixed gently. Flow cytometry was performed using the CytoFLEX (Backman Coulter, CA, USA) and the obtained data were analyzed using CytExpert software version 1.2. The cell size was evaluated by cytometric light scattering of PI-stained cells. To discriminate the live and dead cells, two-parameter histogram was used (FL4LOG vs. FSLOG). Analysis of each sample was performed for 100 sec with high sample delivery. For cell cycle analysis, each cell sample was suspended in 300 μl PBS/serum-free medium containing 200 μg/ml of saponin (Fluka, NY, USA), 250 μg/ml RNase A (Sigma-Aldrich, MO, USA) and 50 μg/ml propidium iodide (PI), incubated for 30 min at a room temperature and subjected to FACS analysis. At least 10,000 cells were measured per sample.

### Measurments of ROS by FACS

For the measurement of intracellular ROS levels, redox-sensitive probe 2′, 7′-dichlorodihydrofluorescein diacetate (H_2_DCFDA, Invitrogen, CA, USA) was used. Cells were loaded with 10 μM H_2_DCFDA and incubated in the dark for 20 min at 37°C, then harvested with tripsin-EDTA solution, suspended in a fresh medium, and immediately analyzed by flow cytometry. The cell fluorescence was measured using flow cytometry, the peak excitation wavelength for oxidized DCF was 488 nm and emission was 525 nm.

### SA-β-Gal activity

Cells expressing senescent-associated β-galactosidase were detected with senescence β-galactosidase staining kit (Cell Signaling Technology, MA, USA) according to manufacturer's instructions. The kit detects β-galactosidase activity at pH 6.0 in cultured cells which is present only in senescent cells and is not found in pre-senescent, quiescent or immortal cells. Quantitative analysis of images presented in Fig. 4c, Suppl. Fig. 1c, Suppl. Fig. 2c was produced with the application of MatLab package, according to the algorithm described in the methodological paper [81]. For each experimental point, it was analyzed not less 250 randomly selected cells.

### Colony forming efficiency essay

Cells were either pretreated or not with 10 μM BAPTA and were subjected to 200 μM H_2_O_2_ for 1 h with the following H_2_O_2_ replacement and cell cultivation under normal conditions for 5 days. Then cells were reseeded onto 6-well plates at an initial density of 1,5*10^2^ per well. After 14 days of cultivation, cells were washed with PBS and stained during 15 min at 37°C with solution, containing 6 % glutaraldehyde, 3 % formaldehyde, 1 % crystal violet (Sigma-Aldrich, MO, USA). Colonies were counted using Adobe Photoshop.

### Western blotting

Western blot analysis was performed as described previously [27]. SDS-PAGE electrophoresis, transfer to nitrocellulose membrane and immunoblotting with ECL (Thermo Scientific, CA, USA) detection were performed according to standard manufacturer's protocols (Bio-Rad Laboratories, PA, USA). Antibodies against the following proteins were used: glyceraldehyde-3-phosphate dehydrogenase (GAPDH) (clone 14C10) (1:1000, #2118S, Cell Signaling, MA, USA), phospho-Rb (Ser807/811) (1:1000, #9308, Cell Signaling, MA, USA), phospho-p53 (Ser15) (clone 16G8) (1:700, #9286, Cell Signaling, MA, USA), p21Waf1/Cip1 (clone 12D1) (1:1000, #2947, Cell Signaling, MA, USA), phospho-ATM (Ser1981) (clone D6H9) (1:1000, #5883, Cell Signaling, MA, USA), anti-phospho-Histone H2A.X (Ser139) (clone JBW301)(1:1000, #05-636, Merck Millipore, Germany), phospho-53BP1 (Ser1778) (1:1000, #2675, Cell Signaling, MA, USA), phospho-AMPKα (Thr172) (clone 40H9) (1:1000, #2535, Cell Signaling, USA), phospho-Tuberin/TSC2 (Ser1387) (1:1000, #5584, Cell Signaling, MA, USA), phospho-p70 S6 Kinase (Thr389) (clone 108D2) (1:1000, #9234, Cell Signaling, MA, USA), phospho-4E-BP1 (Thr37/46) (clone 236B4) (1:1000, #2855, Cell Signaling, MA, USA), LC3 (1:500, #ABC232, Merk KGaA, Germany), phospho-Ulk1 (Ser555) (1:5000, #ABC124, Merk KGaA, Germany), phospho-Ulk1 (Ser757) (1:1000, #6888, Cell Signaling, MA, USA), as well as horseradish peroxidase-conjugated goat anti-rabbit IG (GAR-HRP, #7074S, Cell Signaling, MA, USA) (1:10000) and anti-mouse IG (GAM-HRP, #7076S, Cell Signaling, MA, USA) (1:1000). Hyperfilm (CEA) was from Amersham (Sweden). Equal protein loading was confirmed by Ponceau S (Sigma-Aldrich, MO, USA, #P7170) staining.

### Statistics

Imaging data were analyzed using Mann-Whitney U-test with Bonferroni's correction for multiple comparisons. These results were expressed as means with the standard errors as the error bars. The rest data are presented as the mean and standard deviation of the mean from at least three separate experiments performed and statistical differences were calculated using the Student's t-test. The level of statistical significance was set to p<0.05.

## SUPPLEMENTARY METHODS AND FIGURES



## References

[1] Salido GM (2009). Oxidative Stress, Intracellular Calcium Signals and Apoptotic Processes. Apoptosis: Involvement of Oxidative Stress and Intracellular Ca2+ Homeostasis.

[2] Berridge MJ, Bootman MD, Lipp P (1998). Calcium--a life and death signal. Nature.

[3] Decuypere JP, Bultynck G, Parys JB (2011). A dual role for Ca(2+) in autophagy regulation. Cell Calcium.

[4] Zhivotovsky B, Orrenius S (2011). Calcium and cell death mechanisms: a perspective from the cell death community. Cell Calcium.

[5] Li GY, Fan B, Zheng YC (2010). Calcium overload is a critical step in programmed necrosis of ARPE-19 cells induced by high-concentration H2O2. Biomed Environ Sci.

[6] Vicencio JM, Galluzzi L, Tajeddine N, Ortiz C, Criollo A, Tasdemir E, Morselli E, Ben Younes A, Maiuri MC, Lavandero S, Kroemer G (2008). Senescence, apoptosis or autophagy? When a damaged cell must decide its path--a mini-review. Gerontology.

[7] Kroemer G, Mariño G, Levine B (2010). Autophagy and the integrated stress response. Mol Cell.

[8] McCarthy DA, Clark RR, Bartling TR, Trebak M, Melendez JA (2013). Redox control of the senescence regulator interleukin-1α and the secretory phenotype. J Biol Chem.

[9] Yu X, Li X, Jiang G, Wang X, Chang HC, Hsu WH, Li Q (2013). Isradipine prevents rotenone-induced intracellular calcium rise that accelerates senescence in human neuroblastoma SH-SY5Y cells. Neuroscience.

[10] Farfariello V, Iamshanova O, Germain E, Fliniaux I, Prevarskaya N (2015). Calcium homeostasis in cancer: A focus on senescence. Biochim Biophys Acta.

[11] Ureshino RP, Rocha KK, Lopes GS, Bincoletto C, Smaili SS (2014). Calcium signaling alterations, oxidative stress, and autophagy in aging. Antioxid Redox Signal.

[12] Wiel C, Lallet-Daher H, Gitenay D, Gras B, Le Calvé B, Augert A, Ferrand M, Prevarskaya N, Simonnet H, Vindrieux D, Bernard D (2014). Endoplasmic reticulum calcium release through ITPR2 channels leads to mitochondrial calcium accumulation and senescence. Nat Commun.

[13] Galluzzi L, Vitale I, Kepp O, Kroemer G (2009). Cell Senescence Methods and Protocols.

[14] Fridlyanskaya I, Alekseenko L, Nikolsky N (2015). Senescence as a general cellular response to stress: A mini-review. Exp Gerontol.

[15] Blagosklonny MV (2012). Cell cycle arrest is not yet senescence, which is not just cell cycle arrest: terminology for TOR-driven aging. Aging (Albany NY).

[16] Lecot P, Alimirah F, Desprez PY, Campisi J, Wiley C (2016). Context-dependent effects of cellular senescence in cancer development. Br J Cancer.

[17] Muñoz-Espín D, Serrano M (2014). Cellular senescence: from physiology to pathology. Nat Rev Mol Cell Biol.

[18] Lin J, Yang Q, Wilder PT, Carrier F, Weber DJ (2010). The calcium-binding protein S100B down-regulates p53 and apoptosis in malignant melanoma. J Biol Chem.

[19] Scherz-Shouval R, Weidberg H, Gonen C, Wilder S, Elazar Z, Oren M (2010). p53-dependent regulation of autophagy protein LC3 supports cancer cell survival under prolonged starvation. Proc Natl Acad Sci USA.

[20] Cuervo AM, Bergamini E, Brunk UT, Dröge W, Ffrench M, Terman A (2005). Autophagy and aging: the importance of maintaining “clean” cells. Autophagy.

[21] Gordon PB, Holen I, Fosse M, Røtnes JS, Seglen PO (1993). Dependence of hepatocytic autophagy on intracellularly sequestered calcium. J Biol Chem.

[22] Decuypere JP, Bultynck G, Parys JB (2011). A dual role for Ca(2+) in autophagy regulation. Cell Calcium.

[23] Cárdenas C, Foskett JK (2012). Mitochondrial Ca(2+) signals in autophagy. Cell Calcium.

[24] Patel AN, Park E, Kuzman M, Benetti F, Silva FJ, Allickson JG (2008). Multipotent menstrual blood stromal stem cells: isolation, characterization, and differentiation. Cell Transplant.

[25] Vassena R, Eguizabal C, Heindryckx B, Sermon K, Simon C, van Pelt AM, Veiga A, Zambelli F (2015). and ESHRE special interest group Stem Cells. Stem cells in reproductive medicine: ready for the patient?. Hum Reprod.

[26] Gargett CE, Schwab KE, Deane JA (2016). Endometrial stem/progenitor cells: the first 10 years. Hum Reprod Update.

[27] Borodkina A, Shatrova A, Abushik P, Nikolsky N, Burova E (2014). Interaction between ROS dependent DNA damage, mitochondria and p38 MAPK underlies senescence of human adult stem cells. Aging (Albany NY).

[28] Kawano S, Shoji S, Ichinose S, Yamagata K, Tagami M, Hiraoka M (2002). Characterization of Ca(2+) signaling pathways in human mesenchymal stem cells. Cell Calcium.

[29] Tonelli FM, Santos AK, Gomes DA, da Silva SL, Gomes KN, Ladeira LO, Resende RR (2012). Stem cells and calcium signaling. Adv Exp Med Biol.

[30] Verkhratsky A, Toescu EC (1998). Integrative Aspects of Calcium Signalling.

[31] Decuypere JP, Monaco G, Bultynck G, Missiaen L, De Smedt H, Parys JB (2011). The IP(3) receptor-mitochondria connection in apoptosis and autophagy. Biochim Biophys Acta.

[32] Cárdenas C, Miller RA, Smith I, Bui T, Molgó J, Müller M, Vais H, Cheung KH, Yang J, Parker I, Thompson CB, Birnbaum MJ, Hallows KR, Foskett JK (2010). Essential regulation of cell bioenergetics by constitutive InsP3 receptor Ca2+ transfer to mitochondria. Cell.

[33] Broad LM, Braun FJ, Lievremont JP, Bird GS, Kurosaki T, Putney JW (2001). Role of the phospholipase C-inositol 1,4,5-trisphosphate pathway in calcium release-activated calcium current and capacitative calcium entry. J Biol Chem.

[34] Patterson RL, van Rossum DB, Ford DL, Hurt KJ, Bae SS, Suh PG, Kurosaki T, Snyder SH, Gill DL (2002). Phospholipase C-gamma is required for agonist-induced Ca2+ entry. Cell.

[35] Rogers TB, Inesi G, Wade R, Lederer WJ (1995). Use of thapsigargin to study Ca2+ homeostasis in cardiac cells. Biosci Rep.

[36] Mignen O, Brink C, Enfissi A, Nadkarni A, Shuttleworth TJ, Giovannucci DR, Capiod T (2005). Carboxyamidotriazole-induced inhibition of mitochondrial calcium import blocks capacitative calcium entry and cell proliferation in HEK-293 cells. J Cell Sci.

[37] Burova E, Borodkina A, Shatrova A, Nikolsky N (2013). Sublethal oxidative stress induces the premature senescence of human mesenchymal stem cells derived from endometrium. Oxid Med Cell Longev.

[38] Han X, Tai H, Wang X, Wang Z, Zhou J, Wei X, Ding Y, Gong H, Mo C, Zhang J, Qin J, Ma Y, Huang N (2016). AMPK activation protects cells from oxidative stress-induced senescence via autophagic flux restoration and intracellular NAD(+) elevation. Aging Cell.

[39] Inoki K, Zhu T, Guan KL (2003). TSC2 mediates cellular energy response to control cell growth and survival. Cell.

[40] Roach PJ (2011). AMPK -> ULK1 -> autophagy. Mol Cell Biol.

[41] Wyrsch P, Blenn C, Bader J, Althaus FR (2012). Cell death and autophagy under oxidative stress: roles of poly(ADP-Ribose) polymerases and Ca(2+). Mol Cell Biol.

[42] Qin S, Stadtman ER, Chock PB (2000). Regulation of oxidative stress-induced calcium release by phosphatidylinositol 3-kinase and Bruton's tyrosine kinase in B cells. Proc Natl Acad Sci USA.

[43] Filomeni G, De Zio D, Cecconi F (2015). Oxidative stress and autophagy: the clash between damage and metabolic needs. Cell Death Differ.

[44] Zhang S, Hisatsune C, Matsu-Ura T, Mikoshiba K (2009). G-protein-coupled receptor kinase-interacting proteins inhibit apoptosis by inositol 1,4,5-triphosphate receptor-mediated Ca2+ signal regulation. J Biol Chem.

[45] Pruijn FB, Sibeijn JP, Bast A (1990). Changes in inositol-1,4,5-trisphosphate binding to hepatic plasma membranes caused by temperature, N-ethylmaleimide and menadione. Biochem Pharmacol.

[46] Redondo PC, Salido GM, Rosado JA, Pariente JA (2004). Effect of hydrogen peroxide on Ca2+ mobilisation in human platelets through sulphydryl oxidation dependent and independent mechanisms. Biochem Pharmacol.

[47] Elferink JG (1999). Thimerosal: a versatile sulfhydryl reagent, calcium mobilizer, and cell function-modulating agent. Gen Pharmacol.

[48] Madesh M, Hawkins BJ, Milovanova T, Bhanumathy CD, Joseph SK, Ramachandrarao SP, Sharma K, Kurosaki T, Fisher AB (2005). Selective role for superoxide in InsP3 receptor-mediated mitochondrial dysfunction and endothelial apoptosis. J Cell Biol.

[49] Ermak G, Davies KJ (2002). Calcium and oxidative stress: from cell signaling to cell death. Mol Immunol.

[50] Roveri A, Coassin M, Maiorino M, Zamburlini A, van Amsterdam FT, Ratti E, Ursini F (1992). Effect of hydrogen peroxide on calcium homeostasis in smooth muscle cells. Arch Biochem Biophys.

[51] Az-ma T, Saeki N, Yuge O (1999). Cytosolic Ca2+ movements of endothelial cells exposed to reactive oxygen intermediates: role of hydroxyl radical-mediated redox alteration of cell-membrane Ca2+ channels. Br J Pharmacol.

[52] Rizzuto R (2001). Intracellular Ca(2+) pools in neuronal signalling. Curr Opin Neurobiol.

[53] Bird GS, Burgess GM, Putney JW (1993). Sulfhydryl reagents and cAMP-dependent kinase increase the sensitivity of the inositol 1,4,5-trisphosphate receptor in hepatocytes. J Biol Chem.

[54] Lock JT, Sinkins WG, Schilling WP (2011). Effect of protein S-glutathionylation on Ca2+ homeostasis in cultured aortic endothelial cells. Am J Physiol Heart Circ Physiol.

[55] Khan SR (2013). Reactive oxygen species as the molecular modulators of calcium oxalate kidney stone formation: evidence from clinical and experimental investigations. J Urol.

[56] Görlach A, Bertram K, Hudecova S, Krizanova O (2015). Calcium and ROS: A mutual interplay. Redox Biol.

[57] Foskett JK, White C, Cheung KH, Mak DO (2007). Inositol trisphosphate receptor Ca2+ release channels. Physiol Rev.

[58] Takahashi A, Mikami M, Yang J (2007). Hydrogen peroxide increases GABAergic mIPSC through presynaptic release of calcium from IP3 receptor-sensitive stores in spinal cord substantia gelatinosa neurons. Eur J Neurosci.

[59] de Brito OM, Scorrano L (2010). An intimate liaison: spatial organization of the endoplasmic reticulum-mitochondria relationship. EMBO J.

[60] Pinto MC, Kihara AH, Goulart VA, Tonelli FM, Gomes KN, Ulrich H, Resende RR (2015). Calcium signaling and cell proliferation. Cell Signal.

[61] Takuwa N, Zhou W, Kumada M, Takuwa Y (1993). Ca(2+)-dependent stimulation of retinoblastoma gene product phosphorylation and p34cdc2 kinase activation in serum-stimulated human fibroblasts. J Biol Chem.

[62] Cantoni O, Sestili P, Cattabeni F, Bellomo G, Pou S, Cohen M, Cerutti P (1989). Calcium chelator Quin 2 prevents hydrogen-peroxide-induced DNA breakage and cytotoxicity. Eur J Biochem.

[63] Muehlematter D, Larsson R, Cerutti P (1988). Active oxygen induced DNA strand breakage and poly ADP-ribosylation in promotable and non-promotable JB6 mouse epidermal cells. Carcinogenesis.

[64] Britigan BE, Rasmussen GT, Cox CD (1998). Binding of iron and inhibition of iron-dependent oxidative cell injury by the “calcium chelator” 1,2-bis(2-aminophenoxy)ethane N,N,N',N'-tetraacetic acid (BAPTA). Biochem Pharmacol.

[65] Gunther MR, Hanna PM, Mason RP, Cohen MS (1995). Hydroxyl radical formation from cuprous ion and hydrogen peroxide: a spin-trapping study. Arch Biochem Biophys.

[66] Glickstein H, El RB, Shvartsman M, Cabantchik ZI (2005). Intracellular labile iron pools as direct targets of iron chelators: a fluorescence study of chelator action in living cells. Blood.

[67] Bentle MS, Reinicke KE, Bey EA, Spitz DR, Boothman DA (2006). Calcium-dependent modulation of poly(ADP-ribose) polymerase-1 alters cellular metabolism and DNA repair. J Biol Chem.

[68] Beck C, Robert I, Reina-San-Martin B, Schreiber V, Dantzer F (2014). Poly(ADP-ribose) polymerases in double-strand break repair: focus on PARP1, PARP2 and PARP3. Exp Cell Res.

[69] Bakondi E, Gönczi M, Szabó E, Bai P, Pacher P, Gergely P, Kovács L, Hunyadi J, Szabó C, Csernoch L, Virág L (2003). Role of intracellular calcium mobilization and cell-density-dependent signaling in oxidative-stress-induced cytotoxicity in HaCaT keratinocytes. J Invest Dermatol.

[70] Cuervo AM, Bergamini E, Brunk UT, Dröge W, Ffrench M, Terman A (2005). Autophagy and aging: the importance of maintaining “clean” cells. Autophagy.

[71] Rubinsztein DC, Mariño G, Kroemer G (2011). Autophagy and aging. Cell.

[72] García-Prat L, Martínez-Vicente M, Perdiguero E, Ortet L, Rodríguez-Ubreva J, Rebollo E, Ruiz-Bonilla V, Gutarra S, Ballestar E, Serrano AL, Sandri M, Muñoz-Cánoves P (2016). Autophagy maintains stemness by preventing senescence. Nature.

[73] Bittremieux M, Parys JB, Pinton P, Bultynck G (2016). ER functions of oncogenes and tumor suppressors: modulators of intracellular Ca(2+) signaling. Biochim Biophys Acta.

[74] Chandrachud U, Walker MW, Simas AM, Heetveld S, Petcherski A, Klein M, Oh H, Wolf P, Zhao WN, Norton S, Haggarty SJ, Lloyd-Evans E, Cotman SL (2015). Unbiased Cell-based Screening in a Neuronal Cell Model of Batten Disease Highlights an Interaction between Ca2+ Homeostasis, Autophagy, and CLN3 Protein Function. J Biol Chem.

[75] Høyer-Hansen M, Bastholm L, Szyniarowski P, Campanella M, Szabadkai G, Farkas T, Bianchi K, Fehrenbacher N, Elling F, Rizzuto R, Mathiasen IS, Jäättelä M (2007). Control of macroautophagy by calcium, calmodulin-dependent kinase kinase-beta, and Bcl-2. Mol Cell.

[76] Wang SH, Shih YL, Ko WC, Wei YH, Shih CM (2008). Cadmium-induced autophagy and apoptosis are mediated by a calcium signaling pathway. Cell Mol Life Sci.

[77] Jiang LB, Cao L, Yin XF, Yasen M, Yishake M, Dong J, Li XL (2015). Activation of autophagy via Ca(2+)-dependent AMPK/mTOR pathway in rat notochordal cells is a cellular adaptation under hyperosmotic stress. Cell Cycle.

[78] Cardaci S, Filomeni G, Ciriolo MR (2012). Redox implications of AMPK-mediated signal transduction beyond energetic clues. J Cell Sci.

[79] Sanli T, Steinberg GR, Singh G, Tsakiridis T (2014). AMP-activated protein kinase (AMPK) beyond metabolism: a novel genomic stress sensor participating in the DNA damage response pathway. Cancer Biol Ther.

[80] Zemelko VI, Grinchuk TM, Domnina AP, Artzibasheva IV, Zenin VV, Kirsanov AA, Bichevaia NK, Korsak VS, Nikolsky NN (2012). Multipotent mesenchymal stem cells of desquamated endometrium: Isolation, characterization, and application as a feeder layer for maintenance of human embryonic stem cells. Cell Tissue Biol.

[81] Shlush LI, Itzkovitz S, Cohen A, Rutenberg A, Berkovitz R, Yehezkel S, Shahar H, Selig S, Skorecki K (2011). Quantitative digital in situ senescence-associated β-galactosidase assay. BMC Cell Biol.

