# Supplementary material for: Calcium alterations signal either to senescence or to autophagy induction in stem cells upon oxidative stress
Source: Aging (Albany NY). 2016 Dec 8;8(12):3400–16. doi: 10.18632/aging.101130 (PMC5270676; doi:10.18632/aging.101130)
Supplement: Supplementary file 1 [file aging-08-3400-s001.pdf]

SUPPLEMENTARY MATERIAL

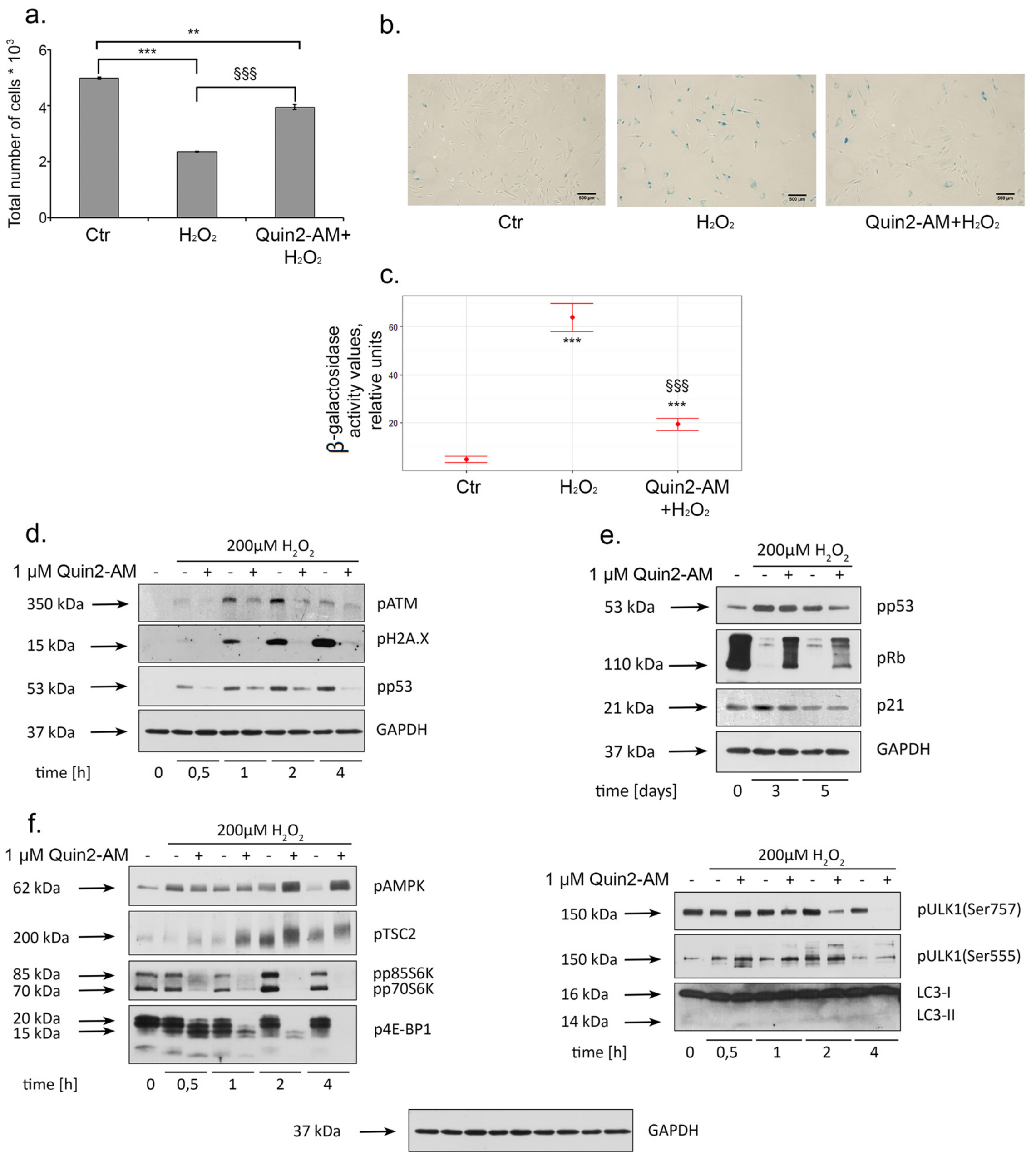

Supplementary Figure 1. Effects of intracellular calcium chelation by Quin2-AM on oxidative stress-induced senescence of hMESCs. Cells were either pretreated or not with 1 µM Quin2-AM (loading procedure is described in “Materials and Methods” section), then were subjected to 200 µM H_2O_2 for 1 h with the following H_2O_2 replacement and cell cultivation under normal conditions for the indicated time. (**a**) Quin2-AM retained cell proliferation as compared to H_2O_2-treated cells. In 5 days after the oxidative stress cells were harvested by trypsinization and plated at a density of $4.5*10^3$ cells per cm^2 and additionally cultured for 5 days. Cell number was determined by FACS. (**b**) SA-β-Gal staining of untreated, H_2O_2-treated and (Quin2-AM+H_2O_2)-treated hMESCs. In 5 days after the oxidative stress cells were harvested by trypsinization and plated at a density of $4.5*10^3$ cells per cm^2 and additionally cultured for 5 days, in order to perform staining in non-confluent cultures. Scale bar is 500 µm and valid for all images. (**c**) Quantification of β-galactosidase activity values in control, H_2O_2-treated and (Quin2-AM+H_2O_2)-treated hMESCs. (**d**) Phosphorylation levels of the main DDR members: ATM, H2A.X, 53BP1, as well as p53. (**e**) Western blot analysis of p53 and Rb phosphorylation, and p21 protein expression performed at indicated time points. (**f**) Western blot analysis of pAMPK, pTSC2, p70S6K and p4E-BP1, pULK1 and LC3 at the various time points after H_2O_2 addition. Representative results of the three experiments are shown in the Figure. GAPDH was used as loading control. Graphs are presented as M ± Std.dev., and the Student’s t-test was used to determine p-value. **$p<0.005$, ***$p<0.001$, versus control; §§§$p<0.001$, versus H_2O_2-treated cells. Ctr – untreated cells.

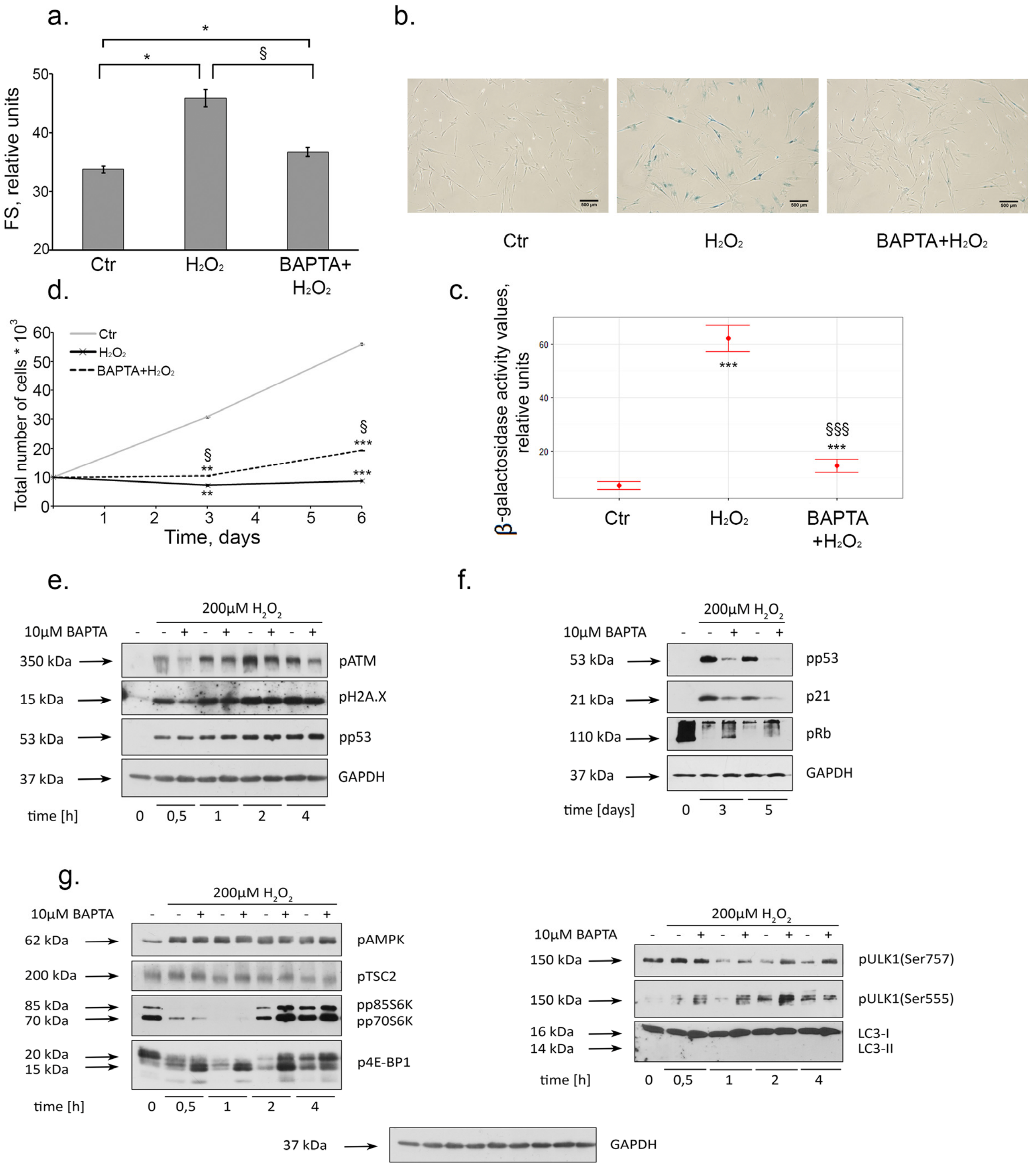

Supplementary Figure 2. Effects of intracellular calcium chelation by BAPTA-AM on oxidative stress-induced senescence of human embryonic fibroblasts. Fibroblasts were treated as indicated in the legend to Figure 3. (**a**) BAPTA partially prevented H_2O_2-induced increase of cell size. Cell size was determined at day 6 after the oxidative stress. Forward scatter (FS) reflects the average cell size. (**b**) SA-β-Gal staining of untreated, H_2O_2-treated and (BAPTA+H_2O_2)-treated fibroblasts. In 5 days after the oxidative stress cells were harvested by trypsinization and plated at a density of $7*10^3$ cells per cm^2 and additionally cultured for 5 days, in order to perform staining in non-confluent cultures. Scale bar is 500 µm and valid for all images. (**c**) Quantification of β-galactosidase activity values in control, H_2O_2-treated and (BAPTA+H_2O_2)-treated fibroblasts. (**d**) BATPA-AM retained cell proliferation as compared to H_2O_2-treated cells. Cell number was determined by FACS at indicated time points. (**e**) Phosphorylation levels of the main DDR members: ATM, H2A.X, 53BP1, as well as p53. (**f**) Western blot analysis of p53 and Rb phosphorylation, and p21 protein expression performed at indicated time points. (**g**) Western blot analysis of pAMPK, pTSC2, p70S6K and p4E-BP1, pULK1 and LC3 at the various time points after H_2O_2 addition. Representative results of the three experiments are shown in the Figure. GAPDH was used as loading control. Graphs are presented as M ± Std.dev., and the Student's t-test was used to determine p-value. *$p<0.05$, **$p<0.005$, ***$p<0.001$, versus control; §$p<0.05$, §§§$p<0.001$, versus H_2O_2-treated cells. Ctr – untreated cells.
